# Supplementary material for: Inhibitor-based modulation of huntingtin aggregation mechanisms mitigates fibril-induced cellular stress
Source: Nat Commun. 2025 Apr 15;16:3588. doi: 10.1038/s41467-025-58691-9 (PMC12000517; doi:10.1038/s41467-025-58691-9)
Supplement: Supplementary file 1 — Supplementary Information [file 41467_2025_58691_MOESM1_ESM.pdf]

Supplementary Information for

Inhibitor-based modulation of huntingtin aggregation mechanisms mitigates fibril-induced cellular stress

Greeshma Jain[+,1], Marina Trombetta-Lima[+,2,3], Irina Matlahov[1], Hennrique Taborda Ribas[2], , Tingting Chen[2], Raffaella Parlato[1], Giuseppe Portale[1], Amalia M. Dolga[2*], Patrick C.A. van der Wel[1*]

* Email: p.c.a.van.der.wel@rug.nl, a.m.dolga@rug.nl

This PDF file includes:

- Supplementary Figures 1-10
- Supplementary Tables 1-5
- Supplementary References

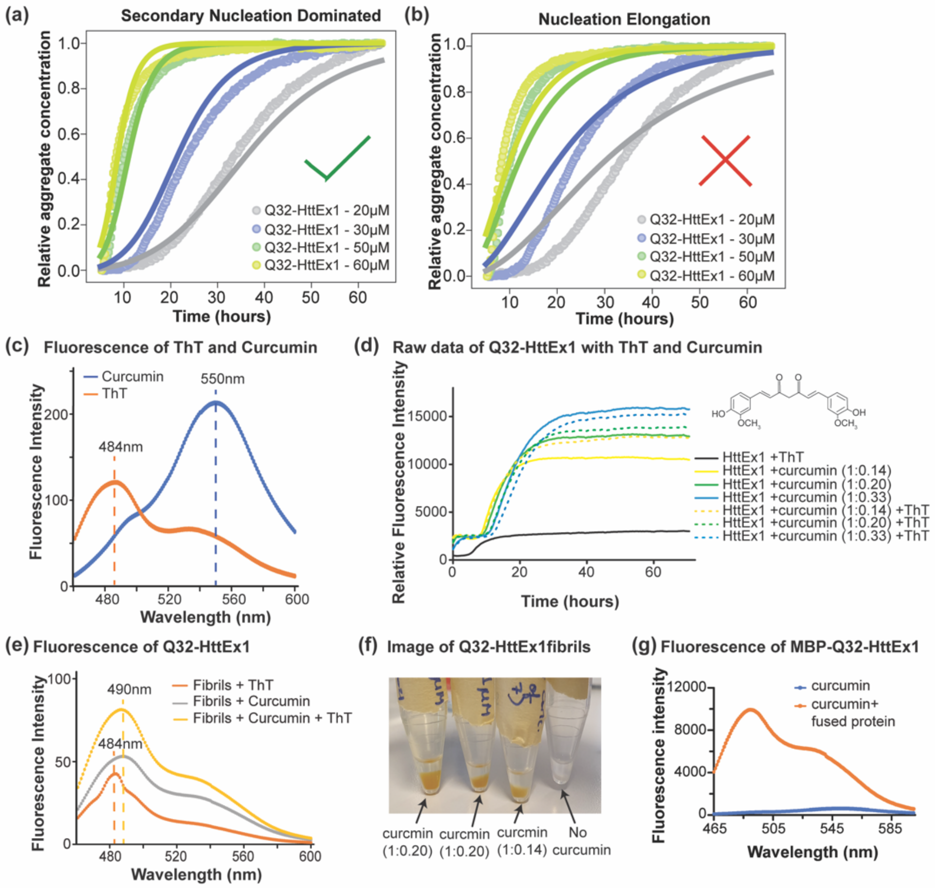

Supplementary Fig. 1. Fluorescence data of ThT and curcumin. (a,b) ThT kinetics data for Q32-HttEx1 aggregation at different concentrations (20μM, 30μM, 50μM and 60μM) were analyzed and plotted in Amylofit (1). Two kinetic models are shown: (a) secondary nucleation dominated, and (b) nucleation elongation (without secondary nucleation). The fitting suggests that the aggregation proceeds predominantly via secondary nucleation. The former mechanism better fits the data. The ThT data were measured in technical triplicates and the mean of the triplicates was plotted. (c) Fluorescence of curcumin and ThT in buffer when excited at 442nm. The dotted lines show the emission maxima for ThT at 484nm and for curcumin at 550nm. (d) Q32-HttEx1 (61μM) aggregation was monitored by ThT fluorescence in the presence and absence of curcumin at various sub-stoichiometric molar ratios, as indicated. Solid lines include samples without ThT, but with curcumin. Dashed lines show the same protein: curcumin ratios with also 15μM ThT present. Note that the fluorescence signal in presence of curcumin is not only showing a delayed increase, but also reaches a much higher fluorescence intensity. The increased intensity stems from the binding of curcumin to the formed HttEx1 fibrils (see panel f), which results in the immobilization of the normally flexible molecule (inset top right) and causes a dramatic increase in curcumin fluorescence. Inclusion of ThT in the sample yields a fluorescent signal intensity that is a combination of the two fluorophores but dominated by the curcumin signal. (e) Fluorescence of Q32-HttEx1 fibrils (30μM) with ThT (15μM) and curcumin (10μM) excited at 442nm. The dotted lines show the emission maxima of ThT bound to fibrils at 484nm and curcumin and ThT bound to the fibrils at 490nm. (f) Photograph of HttEx1 fibrils formed in presence and absence of curcumin, after centrifugation. The fibrils in the pellet display a clear coloring due to curcumin bound to the fibrils. (g) Fluorescence of 10μM curcumin alone compared to that of 10μM curcumin in presence of MBP-Q32-HttEx1 fusion protein (50μM), in PBS and excited at 442nm. Note that the fluorescence intensity units of different panels should not be compared, due to different settings of the spectrofluorometer.

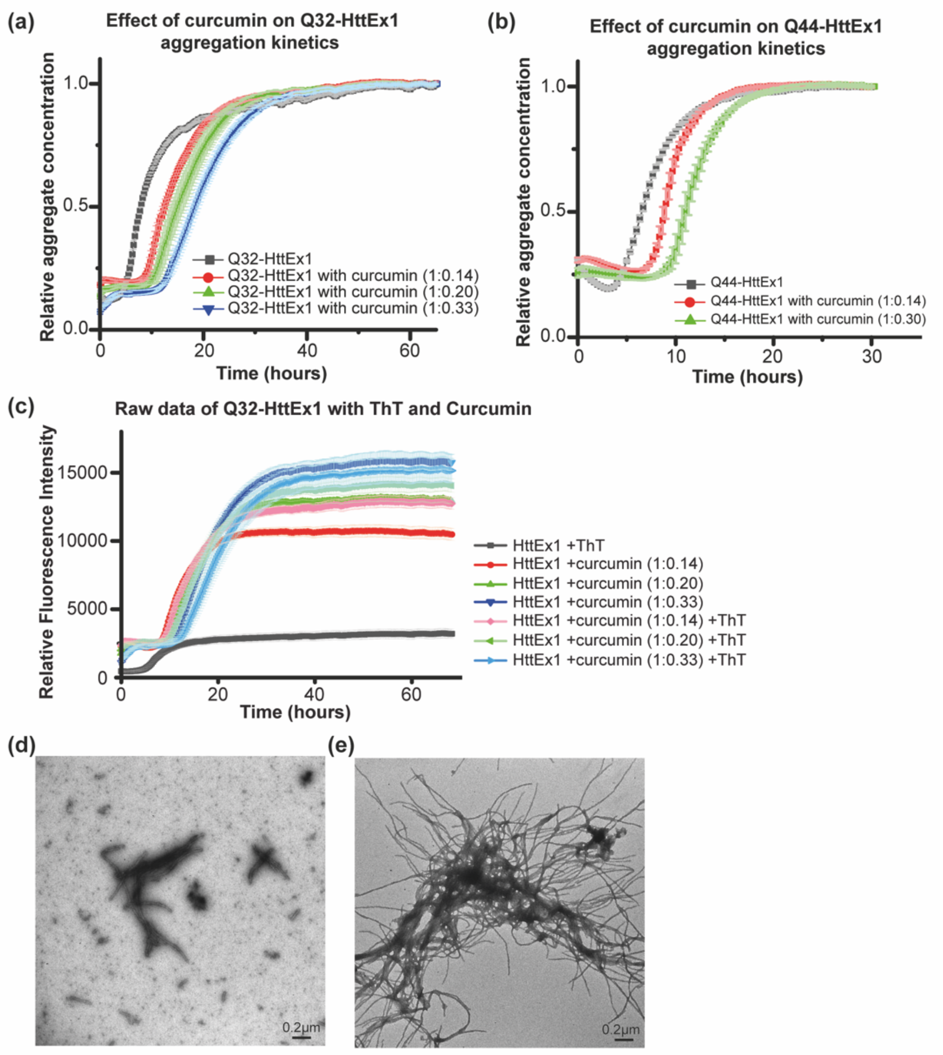

Supplementary Fig. 2. Fluorescence data with error bars and EM analysis showing more bundled fibrils formed in presence of curcumin. (a) Q32-HttEx1 (61μM) aggregation curves (also in figure 2a) measuring fluorescence in the presence and absence of curcumin (molar ratio indicated). (b) Q44-HttEx1 (67.5μM) aggregation fluorescence curves (also in figure 2b) in the presence and absence of curcumin. The curves indicate the mean of triplicate values with error bars (standard deviation). (c) Q32-HttEx1 (61μM) fluorescence aggregation curves (also in Supplementary Figure 1d) in the presence and absence of curcumin at various sub-stoichiometric molar ratios, as indicated. Dark red, green and blue lines include samples without ThT, but with curcumin. Light red, green and blue lines show the same protein:curcumin ratios with also 15μM ThT present. Note that the fluorescence signal in presence of curcumin is not only showing a delayed increase, but also reaches a much higher fluorescence intensity. Inclusion of ThT in the sample yields a fluorescent signal intensity that is a combination of the two fluorophores, but dominated by the curcumin signal. Plotted lines reflect mean of triplicate replicates; error bars reflect standard deviation. (d) Negative stain TEM micrograph of Q32-HttEx1 fibrils prepared *in-vitro* at room temperature. (e) TEM micrograph of Q32-HttEx1 fibrils formed in presence of curcumin (1:0.33), prepared *in vitro* at room temperature. Note the more bundled fibrils in comparison to the fibrils formed in absence of curcumin. These observations are highly reproducible and have been repeated multiple times.

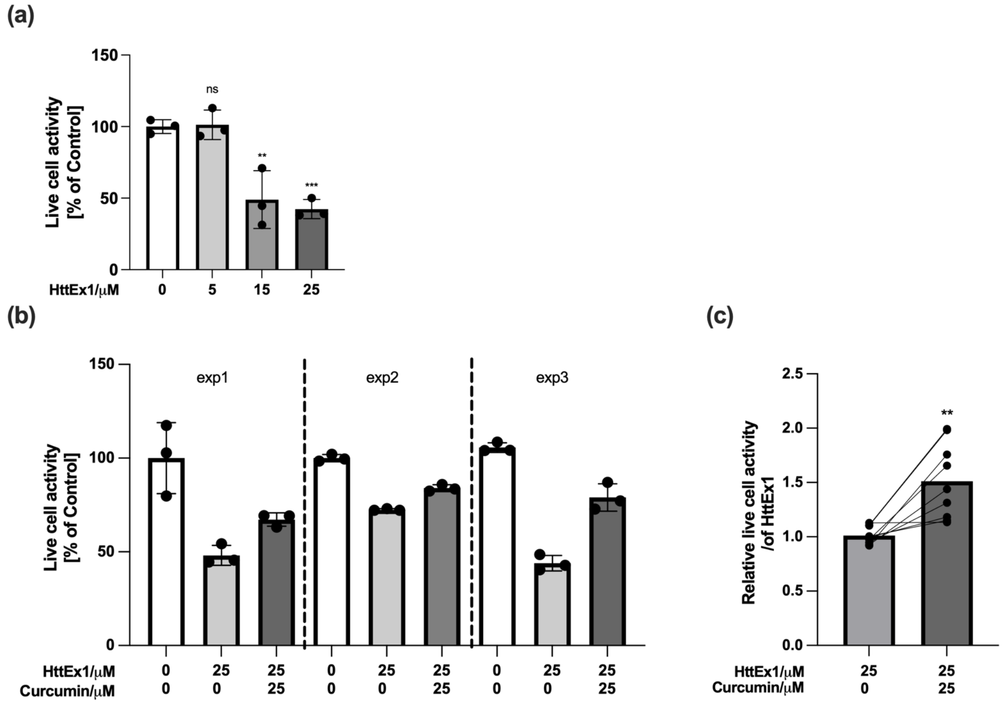

Supplementary Fig. 3. Assays of cytotoxicity caused by HttEx1 fibrils in HT22 cells. (a) Effect of Q32-HttEx1 fibrils on HT22 cells. Live cell activity in HT22 cells was measured using the CytoTox-Glo™ Cytotoxicity Assay, which measures the release of (cytosolic) protease activity from compromised cells, as a measure of cell death. HT22 cells were treated with varying concentrations of Q32-HttEx1 fibrils (5, 15, or 25μM) for 72 hours. Live cell activity was quantified by subtracting the dead cell activity from the total cell activity, measured after cell lysis. These data are obtained from 3 independent experiments with 9 technical replicates each. The error bars indicate mean with SD. Shading reflects protein concentration, as indicated. (b) Effect of curcumin-inhibited Q32-HttEx1 fibrils on treated cells. Live cell activity from three independent experiments (n=3) is shown for HT22 cells treated with 25μM Q32-HttEx1 fibrils or with 25μM Q32-HttEx1 fibrils obtained after curcumin inhibition, for 72 hours. Control samples are cells not exposed to fibrils. Error bars indicate mean with SD. Shading reflects curcumin concentration, as indicated. (c) Relative rescue effect of curcumin inhibition. Relative live cell activity was calculated from data in panel (b), with each dot representing a single technical replicate (9 technical replicates per condition across three biological replicates). Data were normalized to the mean of the 25μM Q32-HttEx1 fibril condition to illustrate the relative rescue effect associated with curcumin inhibition. Shading reflects curcumin concentration, as indicated. Statistical significance was determined using an unpaired t-test ($p < 0.01$).

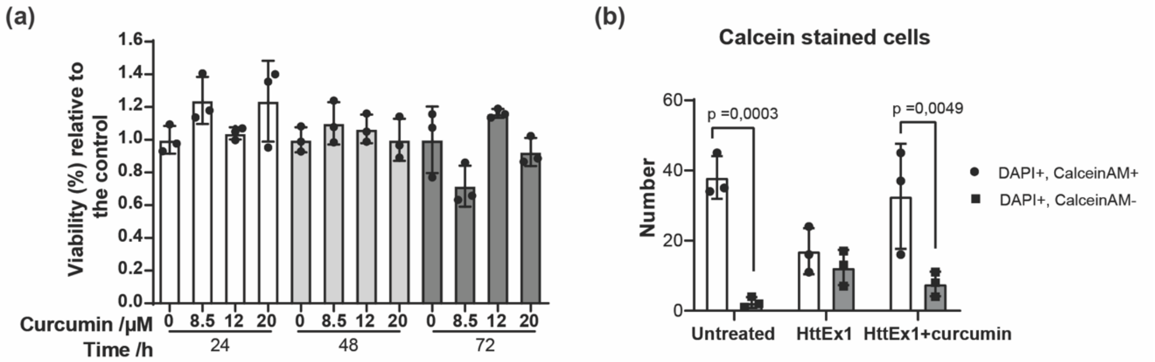

Supplementary Fig. 4. **Effect of curcumin on cell viability and representative graph of cell number under HttEx1 fibrils treatment. (a)** Cell viability of mouse hippocampal HT22 cells upon exposure to varying concentrations of curcumin (in the absence of any HttEx1 fibrils). HT22 cells were seeded in p96 wells at a density of 9.10^3 cells/well. After 24 hours, the cells were treated with 0, 8.5, 12 or 20 µM curcumin diluted in culture medium and incubated for 24, 48 or 72 hours at 37°C and 5% CO_2. Cell viability was assessed through the MTT reduction assay. Absorption values are normalized to the average of the untreated control. One-way ANOVA followed by Benferroni multiple comparisons test. Comparisons were performed against the untreated control for each incubation period. Bars represent the mean ±SD. Shading reflects the incubation time, as indicated (N=3 independent experiments). (b) Differentiated dopaminergic LUHMES cells were exposed for 24 hours to HttEx1 fibrils formed in the absence or presence of curcumin, showing number of cells positive for DAPI and negative for Calcein AM staining; compared to number of cells positive for DAPI and for Calcein AM staining (3 technical replicates per condition). Two-way ANOVA followed by Sidak's multiple comparison test between DAPI+, CalceinAM+ and DAPI+, CalceinAM- groups. Error bars represent the SD (N=3).

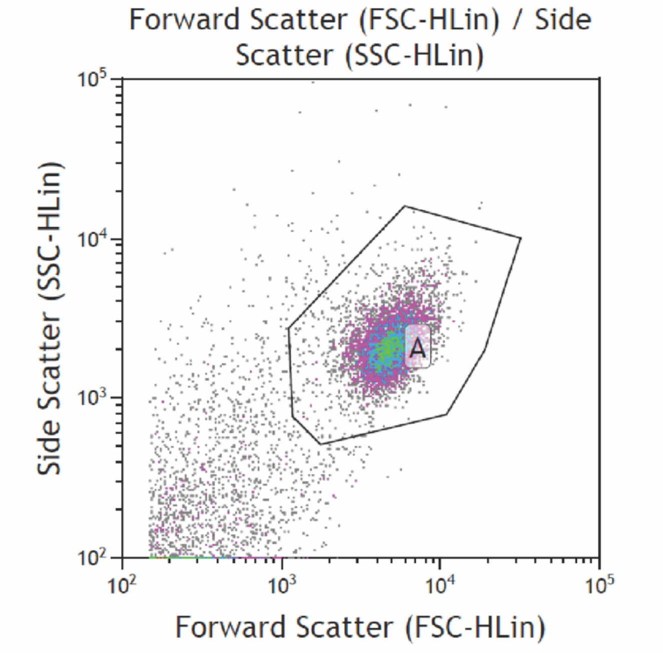

Supplementary Fig. 5. Cytometry gating strategy. A representative image of the density plot of the population of interest. The color scale ranges from grey (low density), pink, blue, green, up to dark red (high density). The selection of the interest population was based on particle size in the SSC versus FSC plot, aiming to exclude debris. The same gate was applied to all conditions and the mean intensity of fluorescence in the FITC channel was evaluated. The population of interest was greater than 75% in all analyzed conditions.

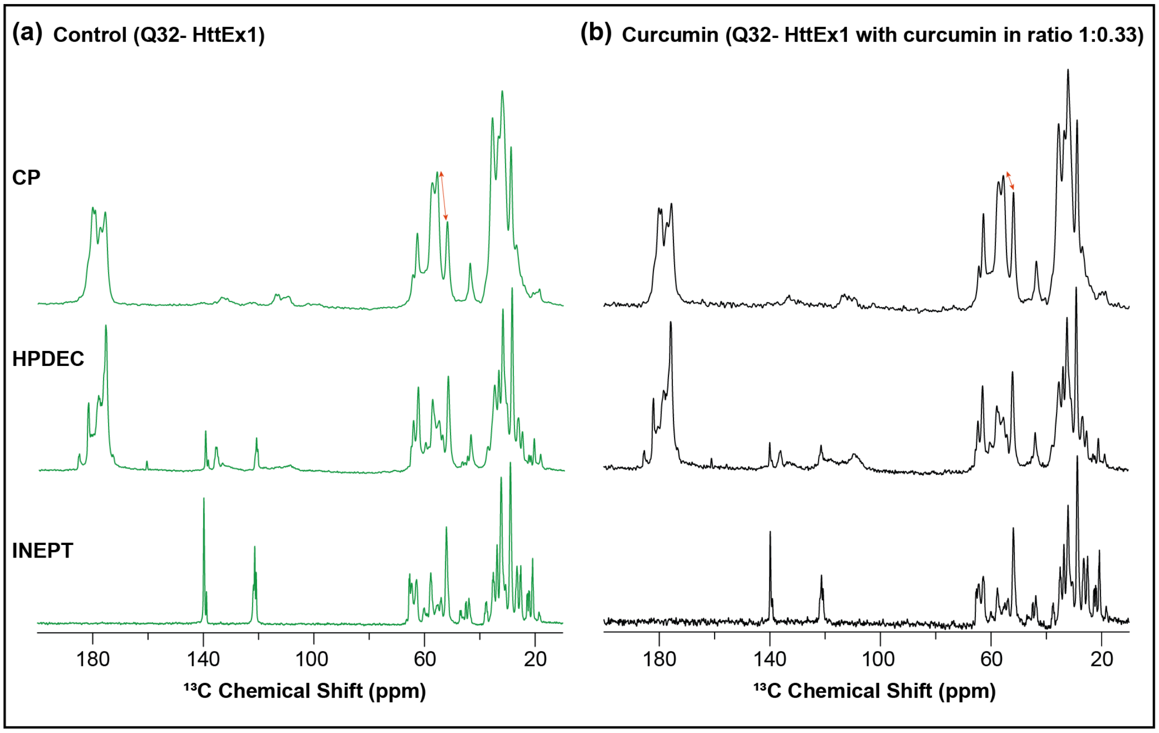

Supplementary Fig. 6. MAS ssNMR comparison of Q32-HttEx1 and Q32-HttEx1 with curcumin (1:0.33) (Batch 1 fibrils). (a) 1D CP ^{13}C spectrum (top), 1D direct excitation (HPDEC) ^{13}C spectrum (middle) and 1D INEPT ^{13}C spectrum (bottom) of Q32-HttEx1 fibrils. (b) 1D CP ^{13}C spectrum (top), 1D HPDEC ^{13}C spectrum (middle) and 1D INEPT ^{13}C spectrum (bottom) of Q32-HttEx1 with curcumin (1:0.33) fibrils. The CP spectra feature signals from rigid and partly immobilized parts of the structure, while the INEPT data show only highly flexible residues. Note the presence of strong signals from the C-terminal His tags in the region of 120-140 ppm (aromatic region) of the INEPT spectra, which shows the highly flexible C-termini being exposed on the fibril surface. The orange arrows in the CP spectra shows the signal intensity between the glutamine and proline signals. Thus, showing the increase in the proline signals for the fibrils formed in presence of curcumin.

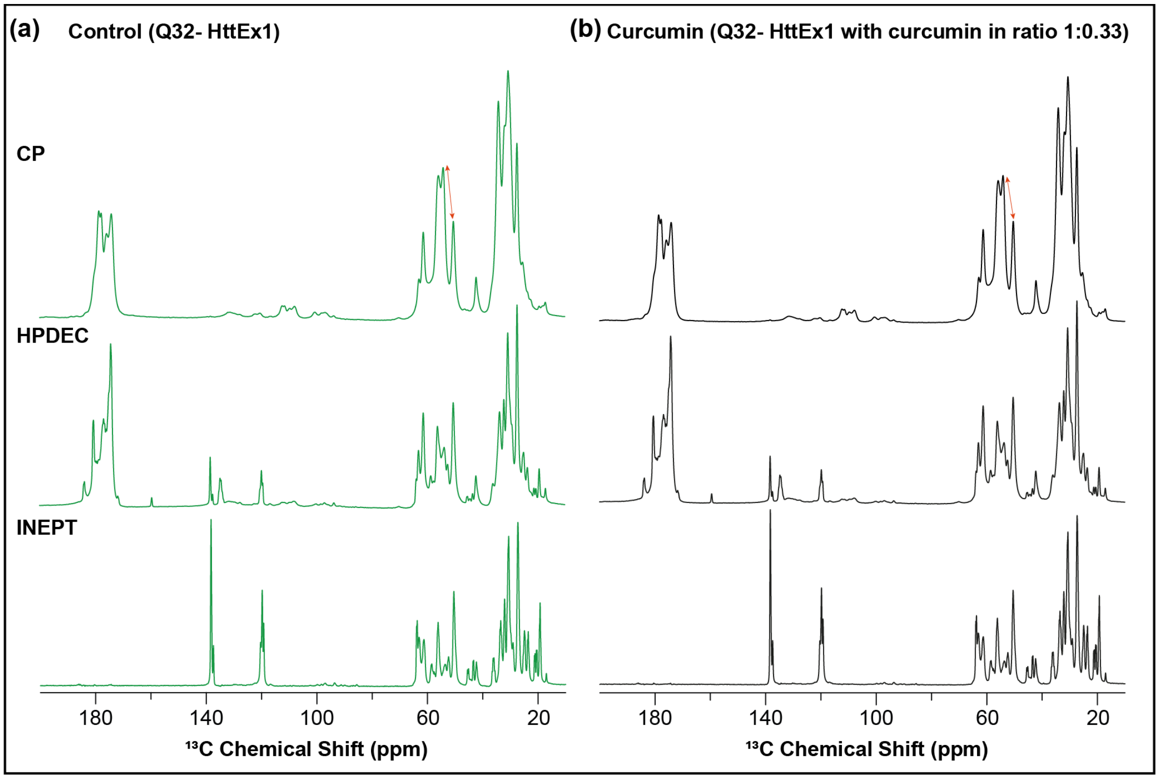

Supplementary Fig. 7. MAS ssNMR comparison of Q32-HttEx1 and Q32-HttEx1 with curcumin (1:0.33) (Batch 2). (a) 1D CP ^{13}C spectrum (top), 1D HPDEC ^{13}C spectrum (middle) and 1D INEPT ^{13}C spectrum (bottom) of Q32-HttEx1 fibrils. (b) 1D CP ^{13}C spectrum (top), 1D HPDEC ^{13}C spectrum (middle) and 1D INEPT ^{13}C spectrum (bottom) of Q32-HttEx1 with curcumin (1:0.33) fibrils. The CP spectra feature signals from rigid and partly immobilized parts of the structure, while the INEPT data show only highly flexible residues. Note the presence of strong signals from the C-terminal His tags in the region of 120-140 ppm (aromatic region) of the INEPT spectra, which shows the highly flexible C-termini being exposed on the fibril surface. The orange arrows in the CP spectra shows the signal intensity differences between the glutamine and proline signals (see also Supplementary Fig. 8 below). Thus, showing the increase in the proline signals for the fibrils formed in presence of curcumin.

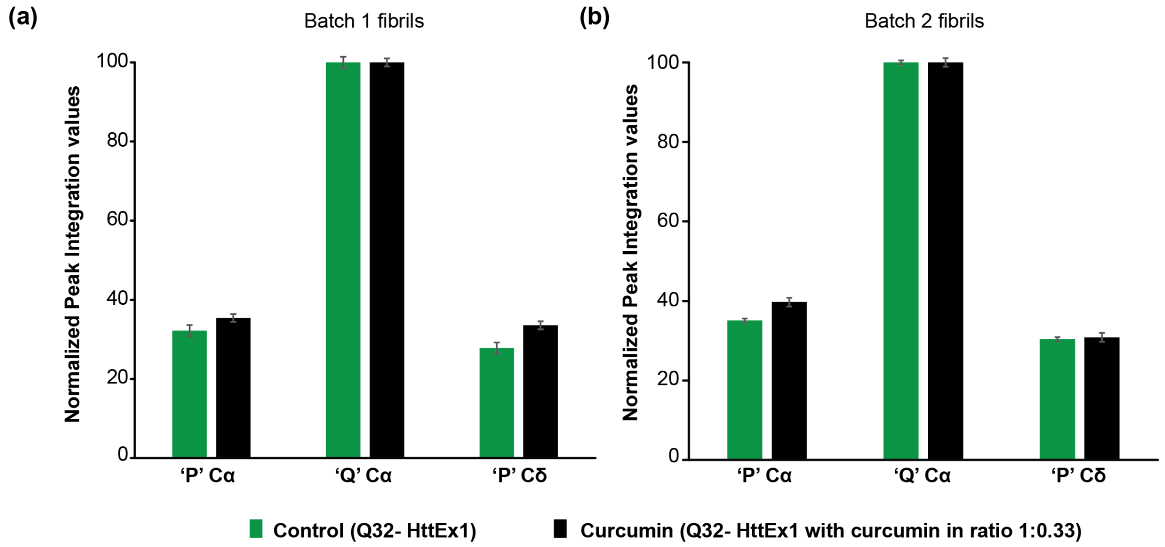

Supplementary Fig. 8. SSNMR peak integration of Proline and Glutamine peaks of Q32-HttEx1 and Q32-HttEx1 with curcumin (1:0.33). Bar graphs representing the normalized peak integration values of the proline and glutamine peaks shown in the ^{13}C CP spectra in (a) supplementary figure 6, batch 1 fibrils and (b) supplementary figure 7, batch 2 fibrils. Normalization of the peak areas was performed relative to the glutamine $C\alpha$ peaks (containing both the a and b type Gln conformers). Green bars correspond to values from the Q32-HttEx1 fibrils and black bars from the Q32-HttEx1 with curcumin (1:0.33). The error bars indicate an estimate of noise in the data. The analysis is based on ssNMR analysis of one sample with curcumin and one sample without curcumin treatment (n=1).

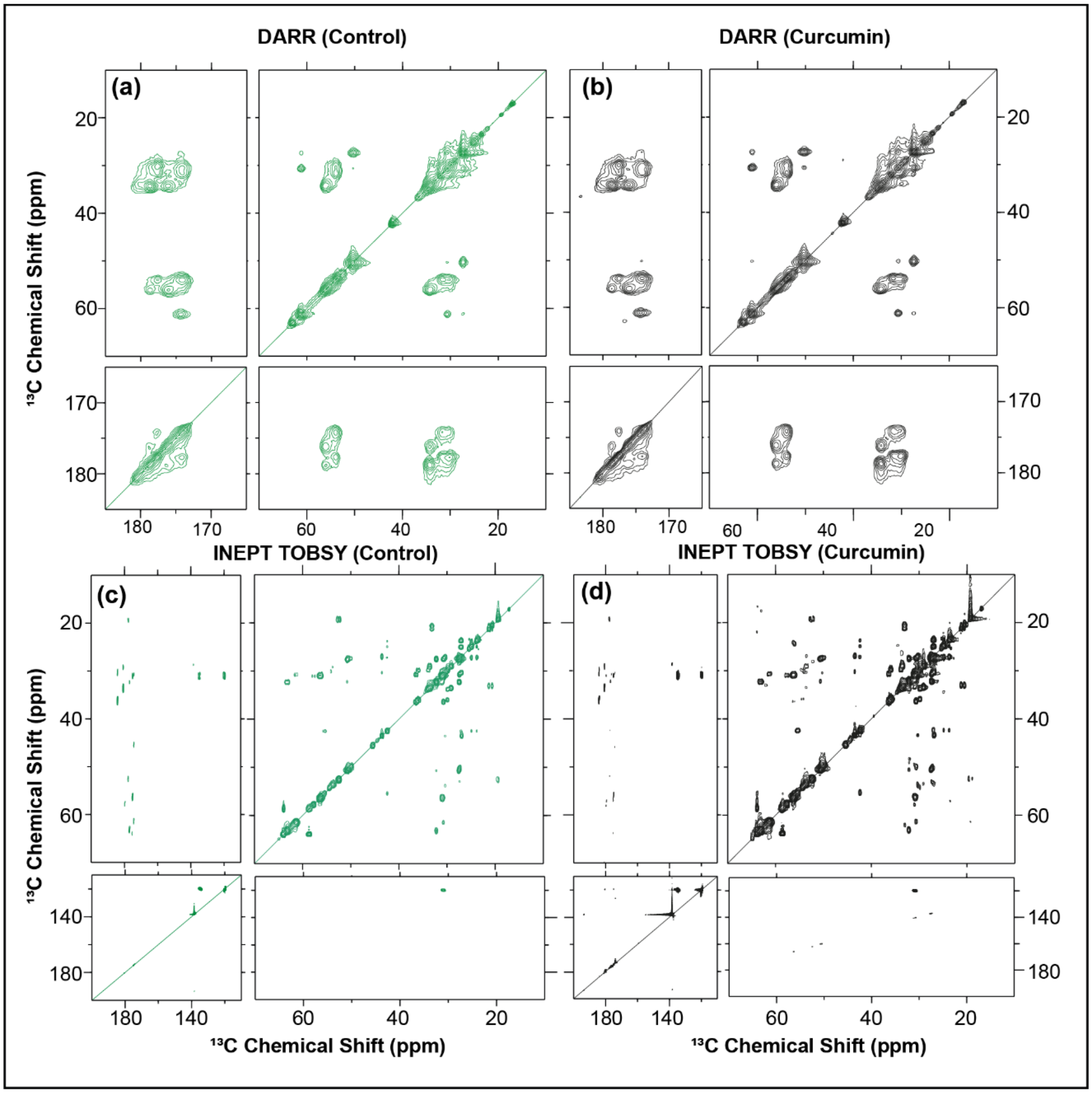

Supplementary Fig. 9. 2D ssNMR analysis of Q32-HttEx1 fibrils formed in presence (black) and absence (green) of curcumin (Batch 2). (a) ^{13}C -^{13}C DARR spectrum for U-^{13}C, ^{15}N Q32-HttEx1 fibrils obtained at 13 kHz MAS and 25ms of DARR mixing. (b) ^{13}C -^{13}C DARR spectrum for U-^{13}C, ^{15}N Q32-HttEx1 fibrils prepared in presence of curcumin (1:0.33) obtained at 13 kHz MAS and 25ms of DARR mixing. (c) ^{13}C -^{13}C INEPT-TOBSY spectrum for U-^{13}C, ^{15}N Q32-HttEx1 fibrils obtained at 10 kHz MAS. (d) ^{13}C -^{13}C INEPT-TOBSY spectrum for U-^{13}C, ^{15}N Q32-HttEx1 fibrils prepared in presence of curcumin (1:0.33) obtained at 10 kHz MAS.

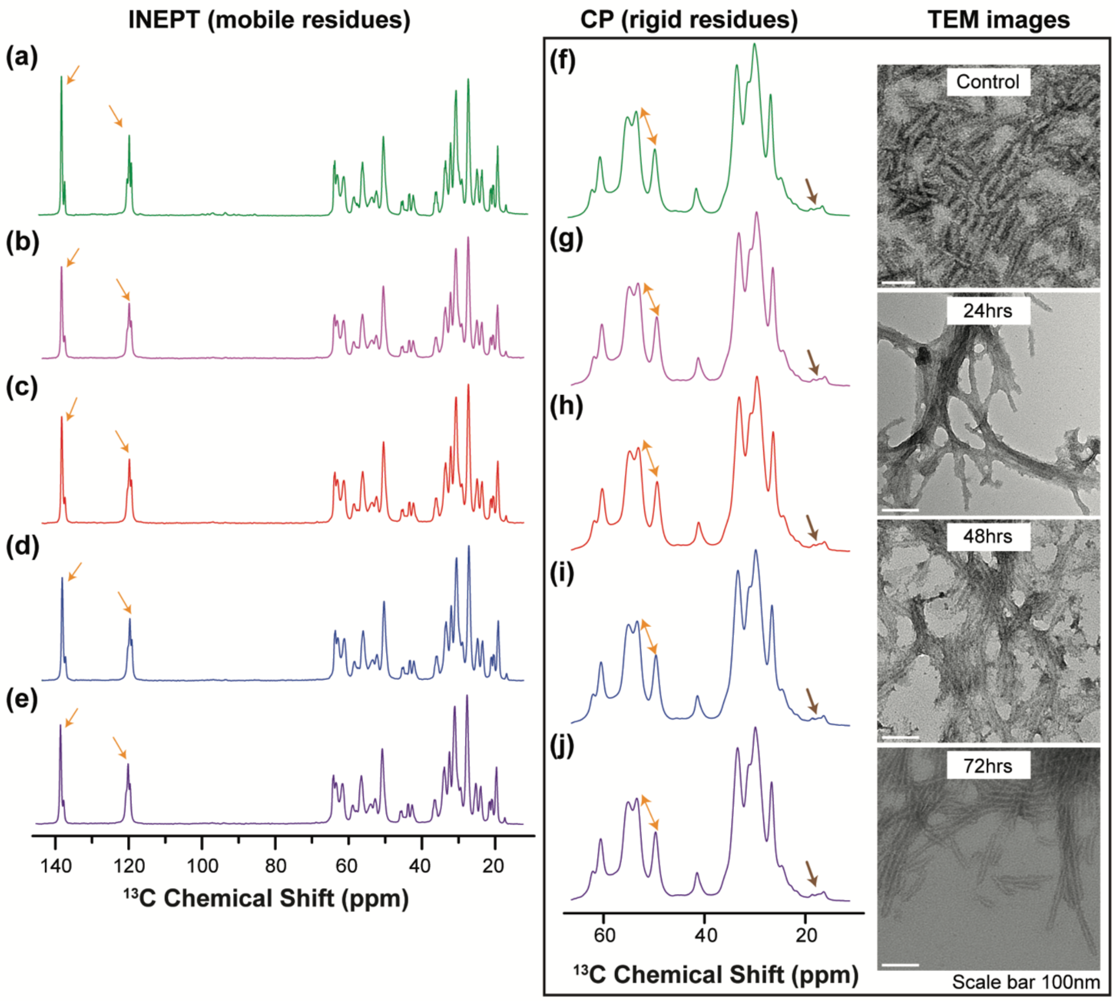

Supplementary Fig. 10. MAS ssNMR and electron microscopy of curcumin addition after aggregation of Q32-HttEx1. 1D INEPT ^{13}C spectra of (a) Q32-HttEx1 fibrils without curcumin, (b) Q32-HttEx1 pre-formed fibrils treated with curcumin (1:0.33) for 24 hours, (c) 48 hours, (d) 72 hours, and (e) 96 hours. Zoomed 1D CP ^{13}C spectra's of (f) Q32-HttEx1 fibrils without curcumin, (g) Q32-HttEx1 pre-formed fibrils treated with curcumin (1:0.33) 24 hours, (h) 48 hours, (i) 72 hours, and (j) 96 hours. TEM images of the pre-formed fibrils with and without treatment with curcumin (1:0.33) are shown on the right. Orange arrows in INEPT spectra indicate residues from the C-terminal tail showing small changes in intensity. The orange arrows in the CP panel mark the signal intensity differences between the glutamine and proline signals (compare to Figure 4). The brown arrows in the CP panel indicate peaks for Ala residues in Htt^{NT} of the HttEx1 fibrils(2).

Supplementary Table 1. Labeling scheme and amount of isotopically labeled MAS ssNMR samples. In all cases, the indicated Q32-HttEx1 proteins were studied as mature amyloid- like fibrils that had been formed at room temperature. The reference to batches indicates two independently prepared samples (see Methods).

Name	Description	Labeling details	Sample size
Sample 1	Q32-HttEx1 fibrils, Batch 1	U- ^{13}C, ^{15}N	2.5mg
Sample 2	Q32-HttEx1 inhibited with curcumin (1:0.33) fibrils, Batch 1	U- ^{13}C, ^{15}N	2.5mg
Sample 3	Q32-HttEx1 fibrils, Batch 2	U- ^{13}C, ^{15}N	15mg
Sample 4	Q32-HttEx1 inhibited with curcumin (1:0.33) fibrils, Batch 2	U- ^{13}C, ^{15}N	20mg
Sample 5	Q32-HttEx1 pre-formed fibrils treated with curcumin (1:0.33)	U- ^{13}C, ^{15}N	11.5mg

Supplementary Table 2. Detailed experimental conditions of the MAS NMR experiments. Abbreviations: NS, number of scans per t_1 point; MAS, magic angle spinning rate; RD, recycle delay; TPPM, ^1H decoupling power during evolution and acquisition (using two-pulse phase modulation scheme); t_1 evolution, number and length (in μs) of t_1 evolution increments. Temperature of the cooling gas for all the experiments was 275K and the mixing time for DARR experiments was 25ms. Sample identification: the sample details are mentioned in Supplementary Table 1 (above).

Figure*	Sample	Experiment	NS	MAS (kHz)	RD (s)	TPPM (kHz)	t_1 evol. (μs)	Contact time (ms)	^{13}C offset (ppm)	^1H offset (ppm)
4(a,b,e) S6(a)	Sample 1	1D ^{13}C CP	1024	10	3	83.3		1	105	3.1
S6(a)	Sample 1	1D ^{13}C INEPT	1024	10	3	50			105	3.5
S6(a)	Sample 1	1D ^{13}C HPDEC	1024	10	3	83.3		1	105	3.1
4(a,b,e) S6(b)	Sample 2	1D ^{13}C CP	1024	10	3	83.3		1	105	3.1
S6(b)	Sample 2	1D ^{13}C INEPT	1024	10	3	50			105	3.5
S6(b)	Sample 2	1D ^{13}C HPDEC	1024	10	3	83.3		1	105	3.1
S7(a), S10 (f)	Sample 3	1D ^{13}C CP	1024	10	3	83.3		1	105	3.3
S7(a), S10 (a)	Sample 3	1D ^{13}C INEPT	1024	10	2.79	50			105	1.3
S7(a)	Sample 3	1D ^{13}C HPDEC	1024	10	3	83.3		1	105	3.3
4c S9(a)	Sample 3	2D DARR	40	13	2.79	83.3	760x36 = 27360		100	-0.2
S9(c)	Sample 3	2D INEPT-TOBSY	32	8.33	3	70	640x30.11= 19270.4		100	-0.2
S7(b)	Sample 4	1D ^{13}C CP	1024	10	3	83.3		1	105	3.3
S7(b)	Sample 4	1D ^{13}C INEPT	1024	10	2.79	50.6			105	1.3
S7(b)	Sample 4	1D ^{13}C HPDEC	1024	10	3	83.3		1	105	3.3
4(d) S9(b)	Sample 4	2D DARR	40	13	2.79	83.3	760x36 = 27360		100	-0.2
S9(d)	Sample 4	2D INEPT-TOBSY	32	8.33	3	72	640x30.11= 19270.4		100	-0.2
S10 (b-e)	Sample 5	1D ^{13}C INEPT	1024	10	3	50			105	1.3
S10 (g-j)	Sample 5	1D ^{13}C CP	1024	10	3	83.3		1	105	3.3

* *Figure identifiers with an S-number refer to figures in the Supplementary Information.*

Supplementary Table 3. SAXS analysis parameters. Fitted parameters using the equation discussed in methods section included in the main manuscript.

	A	σ	$\bar{r}$(nm)	*L** (nm)	*d* (nm)	υ	*B*	*C*	*n*
**Q32-HttEx1**	$6.7e^{-10}$	0.28	6.5	1000	24	100	$2.9e^{-05}$	$1.15e^{-03}$	2.26
**Q32-HttEx1 with curcumin (1:0.33)**	$3.9e^{-09}$	0.35	4.5	1000	20	180	$6.5e^{-04}$	$1.4e^{-03}$	2.16

*this parameter was kept fixed to the mentioned value during the fitting

Supplementary Table 4. Fitted parameters for the SAXS analysis using the model of Perevozchikova et al. (3). Parameters with subscript 2 refer to large objects/aggregates while 1 stands for small objects, the fibrils in our case.

	G_2	R_{g2}*	S_2*	S_1	R_{g1} (nm)	*M*
**Q32-HttEx1**	0.05	1000	1	1	6	3
**Q32-HttEx1 with curcumin (1:0.33)**	0.045	1000	1	1	3.7	3

*these parameters were kept fixed to the mentioned value during the fitting

Supplementary Table 5. Additional SAXS experiment parameters.

(*a*) Sample details		
	Q32 HttEx-1 fibrils	Q32 HttEx-1 fibrils formed with Curcumin
Organism	Human	Human
Source (Catalogue No. or reference)	See Methods	See Methods
Description: sequence (including Uniprot ID + uncleaved tags), bound ligands/modifications, *etc.*	Fibrillized Huntingtin Exon 1, featuring 32 Gln in polyQ segment; with C-terminal HIS tag (Uniprot HTT P42858)	Fibrillized Huntingtin Exon 1, featuring 32 Gln in polyQ segment; with C-terminal HIS tag (Uniprot HTT P42858)
Molecular mass *M* from chemical composition (Da)	12.28kDa	12.28kDa
Concentration measured (mM)	2mM	2mM
Solvent composition	PBS buffer (pH 7.4)	PBS Buffer (pH 7.4)

(*b*) SAXS data collection parameters	
Source, instrument and description	Cu rotating anode from Bruker
Wavelength (Å)	0.105413 nm
Beam geometry (size, sample-to-detector distance)	point-like focusing with 0.3x0.3 microns beamsize, 3.1 m
q-measurement range ($Å^{-1}$ or nm^{-1})	0.05 – 1.4 nm^{-1}
Absolute scaling method	not applicable here
Method for monitoring radiation damage	Constant illumination over 1h to track intensity changes every 10 min. No significant changes detected

Exposure time, number of exposures	10 min single exposure
Sample configuration including path length and flow rate where relevant	1.5 mm glass capillaries with 0.01mm wall thickness
Sample temperature (ºC)	23

(*c*) Software employed for SAXS data reduction, analysis and interpretation

SAXS data reduction: Radial integration to 1D profiles using the Fit2D software. Normalization of the 1D profiles and subtraction using the Origin software.

Data analyses: SasFit software (https://doi.org/10.1107/S1600576722009037)

(d) Shape modelling results: cylindrical objects interacting with PRISM structure factor as presented in the Methods

	Q32 HttEx-1	Q32 HttEx-1 with Curcumin
q-range for fitting	0.05-1.3 nm^{-1}	
Symmetry/anisotropy assumptions	Long cylinder	
χ^2 value/reduced χ^2 value	1368.7/3.7	830.6/2.2
Adjustable parameters in the model fit	Prefactor A, average cylinder cross-sectional radius $\bar{r}$, width of Log-Normal distribution $\sigma(r)$, interaction parameter ν, background parameters (B, C and n)	

(e) Shape modelling results: model presented by Perevozchikova et al. (DOI: 10.1016/j.bpj.2014.06.002) and as described in the Methods

	Q32 HttEx-1 fibrils	Q32 HttEx-1 fibrils formed with Curcumin
q-range for fitting	0.05-1.3 nm^{-1}	
Symmetry/anisotropy assumptions	Generalised Guinier/Porod model	

χ^2 value/reduced χ^2 value	16784.2/45.4	16680.6/45.2
Adjustable parameters in the model fit	Prefactor G2, dimensionality parameter s1, gyration radius of the fibril cross-section Rg1, Porod slope parameter M	

Supplementary References

1. G. Meisl, *et al.*, Molecular mechanisms of protein aggregation from global fitting of kinetic models. *Nat. Protoc.* **11**, 252–272 (2016).
2. H.-K. Lin, *et al.*, Fibril polymorphism affects immobilized non-amyloid flanking domains of huntingtin exon1 rather than its polyglutamine core. *Nat. Commun.* **8**, 15462 (2017).
3. G. Porod, Die Abhangigkeitder Rontgen-Kleinwinkelstreuung von Form und Groβe der kolloiden Teilchen in verdunnten Systemen, IV. *Acta Phys Austriaca* **2**, 255–292 (1948).
