# Supplementary material for: Inhibitor-based modulation of huntingtin aggregation mechanisms mitigates fibril-induced cellular stress
Source: Nat Commun. 2025 Apr 15;16:3588. doi: 10.1038/s41467-025-58691-9 (PMC12000517; doi:10.1038/s41467-025-58691-9)
Supplement: Supplementary file 2 — Reporting Summary [file 41467_2025_58691_MOESM2_ESM.pdf]

nature portfolio

Corresponding author(s): PCA van der Wel, Amalia Dolga

Last updated by author(s): Mar 19, 2025

Reporting Summary

Nature Portfolio wishes to improve the reproducibility of the work that we publish. This form provides structure for consistency and transparency in reporting. For further information on Nature Portfolio policies, see our Editorial Policies and the Editorial Policy Checklist.

Statistics

For all statistical analyses, confirm that the following items are present in the figure legend, table legend, main text, or Methods section.

n/a	Confirmed	
☐	☒	The exact sample size (*n*) for each experimental group/condition, given as a discrete number and unit of measurement
☐	☒	A statement on whether measurements were taken from distinct samples or whether the same sample was measured repeatedly
☐	☒	The statistical test(s) used AND whether they are one- or two-sided<br>*Only common tests should be described solely by name; describe more complex techniques in the Methods section.*
☐	☒	A description of all covariates tested
☐	☒	A description of any assumptions or corrections, such as tests of normality and adjustment for multiple comparisons
☐	☒	A full description of the statistical parameters including central tendency (e.g. means) or other basic estimates (e.g. regression coefficient) AND variation (e.g. standard deviation) or associated estimates of uncertainty (e.g. confidence intervals)
☐	☒	For null hypothesis testing, the test statistic (e.g. *F*, *t*, *r*) with confidence intervals, effect sizes, degrees of freedom and *P* value noted<br>*Give P values as exact values whenever suitable.*
☒	☐	For Bayesian analysis, information on the choice of priors and Markov chain Monte Carlo settings
☒	☐	For hierarchical and complex designs, identification of the appropriate level for tests and full reporting of outcomes
☒	☐	Estimates of effect sizes (e.g. Cohen's *d*, Pearson's *r*), indicating how they were calculated

Our web collection on statistics for biologists contains articles on many of the points above.

Software and code

Policy information about availability of computer code

Data collection	For imaging collection with the Zeiss Cell Discoverer 7, Zen 3.7 software was used. BioTek Gen5 software was used for data collection using Synergy H1 Multi-Mode reader. Cytometry data was collected with CytoExpert v2.<br>The Thioflavin (ThT) kinetics data were collected with Tecan SPARKCONTROL software. The ssNMR data were collected from Bruker Biospin - TopSpin 4.1.3 and processed using NMRPipe version v. 11.5 software.<br>The SAXS experiments were performed at the multipurpose X-ray instrument for nanostructured analysis (MINA). The SAXS patterns have been acquired using a solid-state noiseless Pilatus 300k detector (Dectris) placed 3.1m away from the sample.
Data analysis	FIJI (based on ImageJ2) and more specifically Fiji Macro Plug-In NeurphologyJ were used for image analysis. FlowJo v9.0 and Kaluza Analysis software v2.1 were used for the analysis of cytometry data. Excel v2302 and GraphPad prism v8.0.2 were used for data processing and statistics.<br>The kinetics experiment was done in triplicates and the experiment was repeated. The ThT kinetics data was plotted in Origin 8.1 software. The width analysis data for the TEM images was analyzed using FIJI (based on ImageJ2) software. The ssNMR data was analyzed using the CCPNMR 2.4 software.<br>The 2D SAXS patterns were reduced to the 1D SAXS profiles using the Fit2D and Origin softwares and after proper correction for the difference in sample absorption and subtraction of the background (PBS buffer).

For manuscripts utilizing custom algorithms or software that are central to the research but not yet described in published literature, software must be made available to editors and reviewers. We strongly encourage code deposition in a community repository (e.g. GitHub). See the Nature Portfolio guidelines for submitting code & software for further information.

Data

Policy information about availability of data

All manuscripts must include a data availability statement. This statement should provide the following information, where applicable:

- Accession codes, unique identifiers, or web links for publicly available datasets
- A description of any restrictions on data availability
- For clinical datasets or third party data, please ensure that the statement adheres to our policy

Data Availability
The data generated in this study are available in the figures and supplementary data file, and have been deposited in the Zenodo database under accession code DOI 10.5281/zenodo.14906545 [https://doi.org/10.5281/zenodo.14906545]. Additional data are available from the corresponding authors upon request.

Research involving human participants, their data, or biological material

Policy information about studies with human participants or human data. See also policy information about sex, gender (identity/presentation), and sexual orientation and race, ethnicity and racism.

Reporting on sex and gender	*Use the terms sex (biological attribute) and gender (shaped by social and cultural circumstances) carefully in order to avoid confusing both terms. Indicate if findings apply to only one sex or gender; describe whether sex and gender were considered in study design; whether sex and/or gender was determined based on self-reporting or assigned and methods used.<br>Provide in the source data disaggregated sex and gender data, where this information has been collected, and if consent has been obtained for sharing of individual-level data; provide overall numbers in this Reporting Summary. Please state if this information has not been collected.<br>Report sex- and gender-based analyses where performed, justify reasons for lack of sex- and gender-based analysis.*
Reporting on race, ethnicity, or other socially relevant groupings	*Please specify the socially constructed or socially relevant categorization variable(s) used in your manuscript and explain why they were used. Please note that such variables should not be used as proxies for other socially constructed/relevant variables (for example, race or ethnicity should not be used as a proxy for socioeconomic status).<br>Provide clear definitions of the relevant terms used, how they were provided (by the participants/respondents, the researchers, or third parties), and the method(s) used to classify people into the different categories (e.g. self-report, census or administrative data, social media data, etc.)<br>Please provide details about how you controlled for confounding variables in your analyses.*
Population characteristics	*Describe the covariate-relevant population characteristics of the human research participants (e.g. age, genotypic information, past and current diagnosis and treatment categories). If you filled out the behavioural & social sciences study design questions and have nothing to add here, write "See above."*
Recruitment	*Describe how participants were recruited. Outline any potential self-selection bias or other biases that may be present and how these are likely to impact results.*
Ethics oversight	*Identify the organization(s) that approved the study protocol.*

Note that full information on the approval of the study protocol must also be provided in the manuscript.

Field-specific reporting

Please select the one below that is the best fit for your research. If you are not sure, read the appropriate sections before making your selection.

[x] Life sciences [ ] Behavioural & social sciences [ ] Ecological, evolutionary & environmental sciences

For a reference copy of the document with all sections, see nature.com/documents/nr-reporting-summary-flat.pdf

Life sciences study design

All studies must disclose on these points even when the disclosure is negative.

Sample size	All life science experiments in this study were performed with a minimum of three independent biological replicates to ensure reproducibility and reliability of the results. The chosen sample size aligns with standard practices in the field, allowing for the assessment of biological variability while maintaining feasibility in experimental execution. Although no formal statistical power calculation was performed, the use of at least three independent biological replicates is widely accepted in experimental biology to support robust and interpretable findings. When applicable, statistical analyses were conducted to determine whether observed differences were significant, and variability between replicates was considered in data interpretation.
Data exclusions	For the experiments with eukaryotic cells, no data points were excluded.
Replication	For the assays with eukaryotic cells, each experiment was repeated at least 3 times with different cell passage numbers which was considered a biological replicate. Within each biological replicate, 1 to 6 technical replicates were performed. his information is indicated in each figure legend. Additionally, standard protocols were followed to ensure consistency in sample preparation, data acquisition, and analysis. While

	most findings were successfully replicated, some variability was observed due to differences between fibril batches. This variability is reflected in the data and is inherent to the nature of fibril formation, which can lead to batch-to-batch differences. Despite this, the overall trends remained consistent across experiments, supporting the robustness of our conclusions
Randomization	For all experiments, sample allocation was not random except for microscopy analyses, where image selection and quantification were performed in a blinded manner to minimize observer bias. For flow cytometry cytotoxicity, and MTT assays, the analyses are straightforward and based on objective, automated readouts, reducing the potential for observer bias. In these cases, experimental conditions were predefined based on the study design, ensuring consistency in sample processing and data acquisition.
Blinding	Blinding was applied during microscopy experiments, where image selection and quantification were performed without knowledge of sample identity to minimize observer bias. For flow cytometry and MTT assays, blinding was not implemented as these analyses rely on automated, objective measurements, reducing the potential for subjective influence. Given the straightforward nature of these assays, blinding was not deemed necessary to ensure the integrity of the results.

Reporting for specific materials, systems and methods

We require information from authors about some types of materials, experimental systems and methods used in many studies. Here, indicate whether each material, system or method listed is relevant to your study. If you are not sure if a list item applies to your research, read the appropriate section before selecting a response.

Materials & experimental systems

n/a	Involved in the study
☐	☒ Antibodies
☐	☒ Eukaryotic cell lines
☒	☐ Palaeontology and archaeology
☒	☐ Animals and other organisms
☒	☐ Clinical data
☒	☐ Dual use research of concern
☒	☐ Plants

Methods

n/a	Involved in the study
☒	☐ ChIP-seq
☐	☒ Flow cytometry
☒	☐ MRI-based neuroimaging

Antibodies

Antibodies used	Secondary antibody Alexa Fluor 488 nm Donkey anti-mouse, at a 1:2000 dilution, (Jackson, Cambridgeshire, UK; catalog number 715-545-020) ; anti-THE His Tag (Cat# A00186S, GenScript, New Jersey, US)
Validation	Primary antibody anti-THE: No validation was done by ourselves; supplier Genscript did not specify validation procedures on this particular lot (example applications are available from Genscript DataSheet)

Eukaryotic cell lines

Policy information about cell lines and Sex and Gender in Research

Cell line source(s)	Mouse HT-22 hippocampal cells and Lund human mesencephalic cells (LUHMES) were provided by Prof. Culmsee, University of Marburg, Germany.
Authentication	Cell lines were not specifically authenticated by us.
Mycoplasma contamination	All cells used in this study were regularly tested for mycoplasma contamination. Only cells free from contamination by PCR-based assays were used.
Commonly misidentified lines (See ICLAC register)	None of the cell lines used in this study were reported as commonly misidentified lines in the ICLA register.

Flow Cytometry

Plots

Confirm that:

☒ The axis labels state the marker and fluorochrome used (e.g. CD4-FITC).

☒ The axis scales are clearly visible. Include numbers along axes only for bottom left plot of group (a 'group' is an analysis of identical markers).

☒ All plots are contour plots with outliers or pseudocolor plots.

☒ A numerical value for number of cells or percentage (with statistics) is provided.

Methodology

Sample preparation	HT22 cells were treated with 5, 15 or 20 µM Q32-HttEx1 formed in the absence or presence of curcumin (at a 0.33 ratio) for 24h. Cells were harvested with trypsin and permeabilized or not with 0.1% Triton-X (Sigma) in PBS for 15 min at RT. Next, after 3 washes with PBS, the cells were blocked with 1% BSA (Sigma) in PBS for 1h, followed by incubation with 1µg/mL His-Tag antibody (Cat# A00186S, GenScript, New Jersey, US) for 2h at RT. After 3 washes with PBS, the secondary antibody Alexa Fluor 488 nm Donkey anti-mouse (Jackson, Cambridgeshire, UK) was incubated for 2h, RT. After 3 washes with PBS the cells were resuspended in 300µL PBS.
Instrument	Fluorescence was measured at 690/50 nm in the CytoFLEX benchtop flow cytometer (Beckman Coulter Life Sciences, Indianapolis, US).
Software	Data analysis was performed using FlowJo v9.0 (Becton, Dickinson and Company, Franklin Lakes, US).
Cell population abundance	Mean intensity of fluorescence was analyzed in the total population after the exclusion of debris. The abundance of the population accounted for more than 75% of the total number of detected particles in all conditions and replicates, with values varying from75,41 to 88,26% of total detected particles.
Gating strategy	In order to select the total population of interest, debris was excluded based on the size of the detected particles in the SSC versus FSC plot. The mean intensity of fluorescence was evaluated in the resulting population.

☒ Tick this box to confirm that a figure exemplifying the gating strategy is provided in the Supplementary Information.
